# Identification of LTF as a Prognostic Biomarker for Osteosarcoma

**DOI:** 10.1155/2022/4656661

**Published:** 2022-01-21

**Authors:** Xiaoqi Liu, Zengqiang Wang, Meijiao Liu, Fengnan Zhi, Pengpeng Wang, Xingyu Liu, Shanxiao Yu, Bing Liu, Yanan Jiang

**Affiliations:** ^1^Department of Orthopedic Surgery, The Second Affiliated Hospital, Harbin Medical University, Harbin, China; ^2^Department of Pharmacy, Anqiu People's Hospital, Anqiu, China; ^3^Department of Oral and Maxillofacial Surgery, The First Affiliated Hospital, Harbin Medical University, Harbin, China; ^4^Department of Pharmacology (State-Province Key Laboratories of Biomedicine-Pharmaceutics of China, Key Laboratory of Cardiovascular Research, Ministry of Education), College of Pharmacy, Harbin Medical University, Harbin, China; ^5^Continuing Education Office, The Second Affiliated Hospital, Harbin Medical University, Harbin, China; ^6^Academic Affairs Office, The Second Affiliated Hospital, Harbin Medical University, Harbin, China; ^7^College of Humanities and Social Sciences, Harbin Medical University, Harbin, China; ^8^Translational Medicine Research and Cooperation Center of Northern China, Heilongjiang Academy of Medical Sciences, Harbin, China

## Abstract

Osteosarcoma remains a major health problem in teenagers. However, its pathogenesis mechanism remains not fully elucidated. This study aims to identify the prognostic biomarkers for osteosarcoma. In this study, we selected genes with a median absolute deviation (MAD) value of the top 5000 in the GSE32981 dataset for subsequent analysis. Weighted correlation network analysis (WGCNA) was used to construct a coexpression network. WGCNA showed that the tan module and midnight blue module were highly correlated with origin and metastases of osteosarcoma, respectively. Enrichment analysis was conducted using genes in the tan module and midnight blue module. A gene coexpression network was constructed by calculating the Spearman correlation coefficients. Four key genes (LTF, C10orf107, HIST1H2AK, and NEXN) were identified to be correlated with the prognosis of osteosarcoma patients. LTF has the highest AUC value, and its effect on osteosarcoma cells was then evaluated. The effect of LTF overexpression on proliferation, migration, and invasion of MG63 and 143B cells was detected by the CCK-8 assay, transwell cell migration assay, and transwell invasion assay, respectively. The overexpression of LTF promoted the proliferation, migration, and invasion of MG63 and 143B cells. In conclusion, LTF may serve as a prognostic biomarker for osteosarcoma.

## 1. Introduction

Osteosarcoma is a kind of primary malignant bone tumors that prefer to occur in teenagers. It is estimated to occur in 2% and 3% cancer cases in age at birth to 14 and 15 to 19, respectively [1]. Despite the advances in therapy, the 5-year survival rate of osteosarcoma was only about 67%∼69% [1]. Therefore, osteosarcoma remains a major health problem. The identification of novel biomarkers for osteosarcoma is still needed. Finding more accurate biomarkers would promote the outcome prediction and individualized therapy of osteosarcoma patients.

With the development of high-throughput detection techniques, a large number of genes have been found to be differentially expressed in osteosarcoma [2]. Namløs et al. performed microarray analysis and identified gene expression profiles in osteosarcoma samples of primary and metastatic origin [3]. Buddingh et al. identified gene signatures related to the metastases of osteosarcoma patients [4]. Some differentially expressed genes have been identified as biomarkers for osteosarcoma. For example, CBX3 and ABCA5 are identified as putative biomarkers for tumor stem cells in osteosarcoma [5]. High MMP9 expression was associated with poor overall survival of osteosarcoma patients [6]. Myc was highly expressed in human osteosarcoma cell lines and tissues. Higher Myc expression was correlated with metastasis and poor prognosis of patients with osteosarcoma [7]. However, the effect of altered genes in osteosarcoma has still not been fully addressed. Also, there is still a lack of understanding in the relationships among these genes.

Weighted gene coexpression network analysis (WGCNA) could find clusters (modules) of highly correlated genes [8]. WGCNA has been applied to screening biomarkers and drug targets in various cancers, including lung cancer [9], colon cancer [10], and bladder cancer [11]. This method could be also used in screening biomarkers for osteosarcoma.

In this study, osteosarcoma-related high-throughput data were retrieved from the Gene Expression Omnibus (GEO) database (https://www.ncbi.nlm.nih.gov/geo/). The data were analyzed using bioinformatic methods. We screened 326 candidate genes related to the origin and metastasis of osteosarcoma by WGCNA. Subsequently, we identified 4 key genes correlated with the survival of osteosarcoma patients, including lactoferrin/lactotransferrin (LTF), C10orf107, histone cluster 1 H2ak (HIST1H2AK), and nexilin F-actin binding protein (NEXN). Furthermore, the effect of LTF on proliferation, migration, and invasion of osteosarcoma cells was confirmed.

## 2. Material and Methods

### 2.1. Gene Expression Data Collection

We searched the osteosarcoma-related datasets in the GEO database (https://www.ncbi.nlm.nih.gov/gds/). Two datasets were involved in this work. The dataset GSE32981 from GPL3307 was used as the training dataset. The dataset GSE21257 from the GPL10295 platform was used as the testing dataset.

### 2.2. WGCNA

The median absolute deviation (MAD) value was used to screen the top 5000 genes from the GSE32981 dataset. A scale-free coexpression network was established using the WGCNA package in R software [8]. The gene modules were identified by calculating the topological overlap matrix (TOM). Metascape (https://metascape.org/) was used to perform enrichment analysis [12].

### 2.3. Coexpression Network Establishment

Spearman correlation coefficient (SCC) values of gene pairs were calculated. Gene pairs with |SCC| > 0.6 were involved in the coexpression network. Cytoscape software was used to screen the top ten nodes with the highest degree in tan and midnight blue modules, respectively.

### 2.4. The Prognostic Value of Genes

The prognostic value of hub nodes in the constructed coexpression network was calculated. Kaplan–Meier analysis of these hub nodes was conducted using data in GSE21257. Receiver operating characteristic (ROC) curves are used to describe the prediction accuracy of the hub nodes. The area under the ROC curve (AUC) was analyzed.

### 2.5. Cell Culture and Transfection

MG63 and 143B cells were cultured in MEM medium (Gibco, Cat. No. C11095500BT) with FBS (Hyclone, Cat. No. SH30087.01) and penicillin-streptomycin (Hyclone, Cat. No. SH30010). These two cell lines were incubated under 5% CO_2_ at 37°C.

The overexpression plasmids containing the whole coding sequence of LTF with the Flag-tag and pcDNA3.1 vector served as the negative control. Real-time PCR product sequencing was conducted to confirm that the LTF sequence was successfully cloned into pcDNA3.1 vector. MG63 and 143B cells were cultured with a confluence of 70%–80% and transfected with Lipofectamine 2000 reagent (Invitrogen, Cat. No. 11668019) with the plasmid.

### 2.6. CCK-8 Assay

The proliferation of MG63 and 143B cells was detected before and 1, 2, 3, and 4 days after transfection using a CCK-8 assay kit (Jiangsu KeyGEN BioTECH Co. Ltd, Cat. No. KGA317). MG63 and 143B cells were cultured with the CCK-8 reagent for 4 h, and then the optical density was detected at 450 nm by using a microplate reader (Thermo Fisher Scientific, Multiscan MK3).

### 2.7. Cell Migration and Invasion Assay

The migration and invasion ability of MG63 and 143B cells was detected by the transwell cell migration assay and transwell cell invasion assay, respectively. The difference between the two experiments is that, for the transwell cell invasion assay, the upper chamber was coated with Matrigel (BD Biosciences). In detail, the upper chambers of the transwell plate (BD Biosciences) contain a culture medium without FBS, and the lower chambers contain a complete culture medium. Forty-eight hours after incubation, invasion cells were fixed with 4% paraformaldehyde and stained with crystal violet. After washed with PBS, the images of cells were captured. Data were analyzed using ImageJ software.

### 2.8. Statistical Analysis

Statistical analysis was conducted using GraphPad Prism 8 and R version 3.6.1. Data were presented as mean ± standard deviation. Comparison between two groups was performed using Student's *t*-tests. *P* < 0.05 was considered statistically significant.

### 2.9. Ethics Statement

This work did not involve human participants or animals.

## 3. Results

### 3.1. Clustering of Coexpression Module Eigengenes in Osteosarcoma

**Figure 1:**
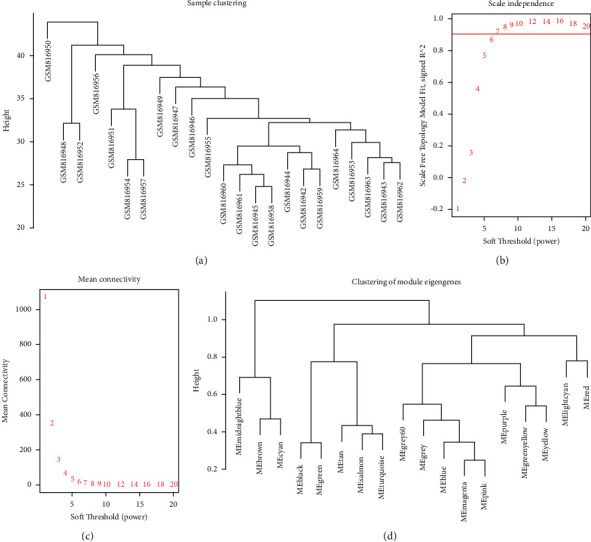
The WGCNA of potential biomarkers for osteosarcoma. (a) Sample clustering of GSE32981 to detect outliers. (b) The correlation coefficients between log (*K*) and log (*P*(*k*)) corresponding to different soft thresholds. (c) A genetic network corresponding to different soft thresholds. (d) Sample cluster of module eigengenes.

Genes in the GSE32981 dataset with the top 5000 MAD values were used for WGCNA analysis. There were no discrete samples as revealed by clustering of the samples (Figure 1(a)). Next, to make the constructed network conform to the characteristics of the scale-free network, we performed the screening with a soft threshold. In this study, the scale-independent value reached 0.9 when the power value *β* was set to 7, and the average connectivity ratio was low (Figures 1(b) and 1(c)). Therefore, the coexpression matrix was calculated under the condition of determined *β* = 7. The cluster of module eigengenes is shown in Figure 1(d).

### 3.2. Identification of Key Modules in Osteosarcoma

**Figure 2:**
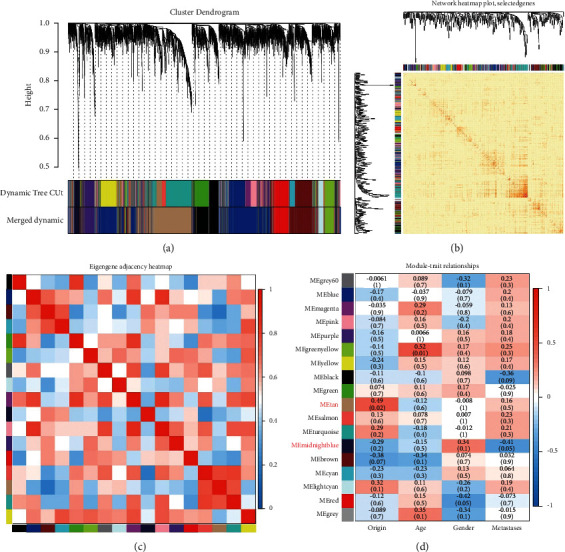
Identification of modules and key genes related to osteosarcoma. (a) Gene cluster tree classification diagram. (b) Heatmap plot of topological overlap in the gene network. (c) The eigengene of each colored module was calculated and an adjacency matrix was established. (d) The relevance between eigengenes of 18 modules and status of osteosarcoma samples.

Similar clusters were combined into a new module using two settings: height = 0.2 and min module size = 50. Eighteen modules with similar patterns of connected genes were obtained (Figure 2(a)). Through the correlation study of the network heatmap plot (Figure 2(b)), there was little correlation among the 18 modules. The adjacency relationship between gene and gene in the module is shown in Figure 2(c). The relevance between the 18 modules and characteristics (origin, age, gender, and metastases) of osteosarcoma samples was evaluated based on module-trait relationships (MTRs). The tan module included 195 genes, which showed the highest positive correlation with origin. The midnight blue module included 131 genes, which showed the highest negative correlation with metastases (Figure 2(d)). The tan module was correlated with cancer origin (primary or metastasis). The midnight blue module was correlated with cancer metastasis. Therefore, the tan module and midnight blue module were selected for further analyses.

### 3.3. Enrichment Analysis of Genes Involving in Tan Module and Midnight Blue Module

**Figure 3:**
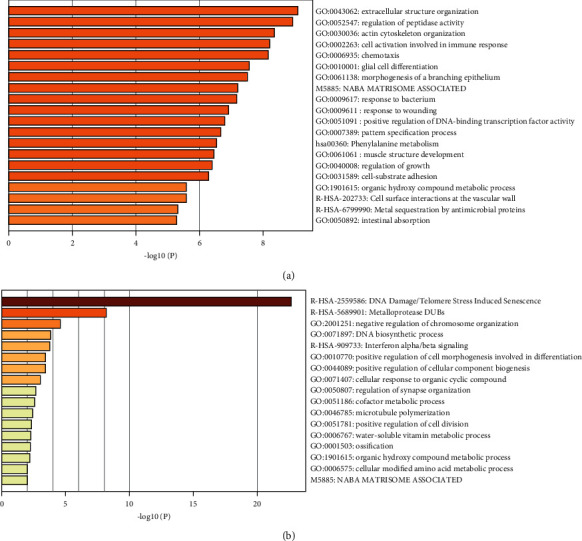
Functional enrichment of genes involving in the tan module and midnight blue module. (a) Functional enrichment of genes involving in the tan module. (b) Functional enrichment of genes involving in the midnightblue module.

The enrichment analysis was performed using genes involving in the tan module and midnight blue module, respectively. The results showed that genes involving in the tan module were mainly enriched in biological processes including “extracellular structure organization,” “regulation of peptidase activity,” and “actin cytoskeleton organization” (Figure 3(a)). Also, genes involving in the midnight blue module were mainly enriched in biological processes including “DNA damage/telomere stress-induced senescence,” “metalloprotease DUBs,” and “negative regulation of chromosome organization” (Figure 3(b)).

### 3.4. Coexpression Network of Genes Involving in the Modules

**Figure 4:**
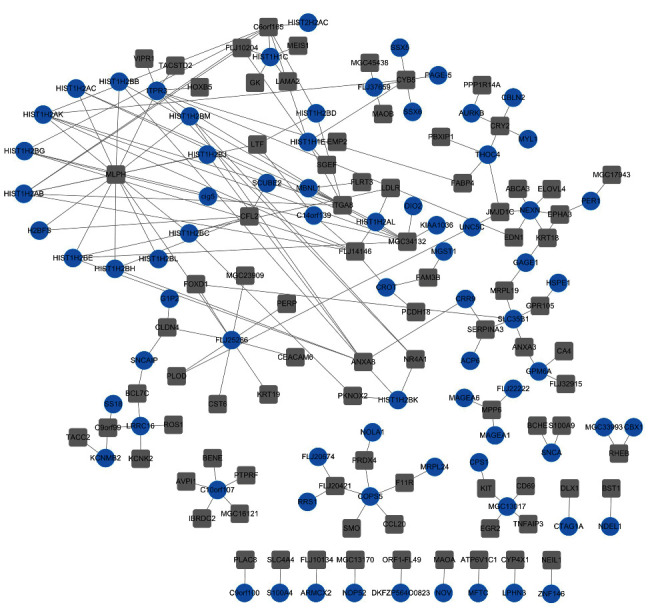
Coexpression network of genes involving in the tan module and midnightblue module. Square gray and circular blue nodes represent genes involving in the tan module and midnightblue module, respectively.

A coexpression network was constructed using genes involving in the tan module and midnight blue module based on the gene coexpression relationship. Spearman correlation coefficient was calculated using genes in the tan module and midnight blue module. 177 gene pairs with |SCC| > 0.6 were screened to construct the coexpression network, including 160 nodes and 177 edges (Figure 4).

### 3.5. Validation of Key Genes in the Coexpression Network

**Figure 5:**
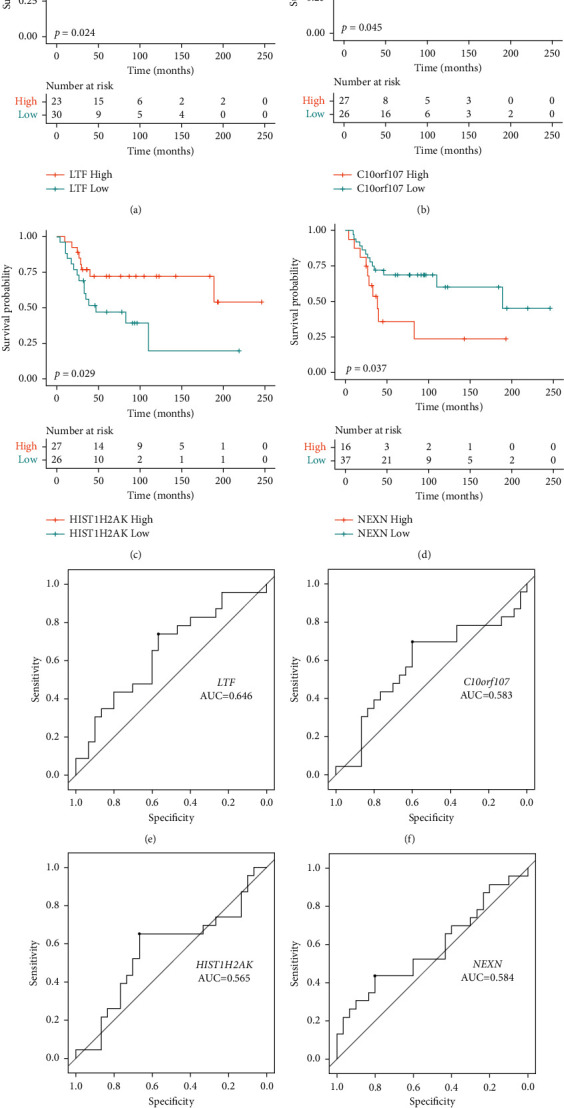
Kaplan–Meier survival curve and ROC curve of key genes. Kaplan–Meier survival cures for LTF (a), C10orf107 (b), HIST1H2AK (c), and NEXN (d), ROC curve of LTF (e), C10orf107 (f), HIST1H2AK (g), and NEXN (h).

Subsequently, we analyzed the topological properties of the established coexpression network. The top 10 hub nodes with high degree involved in the tan module and midnight blue module are shown in Tables 1 and 2, respectively. Kaplan–Meier analysis of these key genes was performed using the GSE21257 dataset. LTF in the tan module was correlated with the prognosis of osteosarcoma patients (Figure 5(a)). C10orf107, HIST1H2AK, and NEXN in the midnightblue module were correlated with the prognosis of osteosarcoma patients (Figures 5(b)–5(d)). The ROC of specificity and sensitivity was analyzed, and the area under the curve (AUC) was then computed. The AUC of LTF, C10orf107, HIST1H2AK, and NEXN was 0.646, 0.583, 0.565, and 0.584, respectively (Figures 5(e)–5(h)). Among these genes, the AUC of LTF was the highest, which indicates its effectiveness as a prognostic biomarker in osteosarcoma. In addition, two independent datasets (GSE36001 and GSE99671) from the GEO database were used to compare the LTF expression in osteosarcoma samples and normal controls. The results showed that the expression of LTF was lower in osteosarcoma samples compared with that in normal controls (Figure S1).

### 3.6. The Effect of LTF on Osteosarcoma Cells

**Figure 6:**
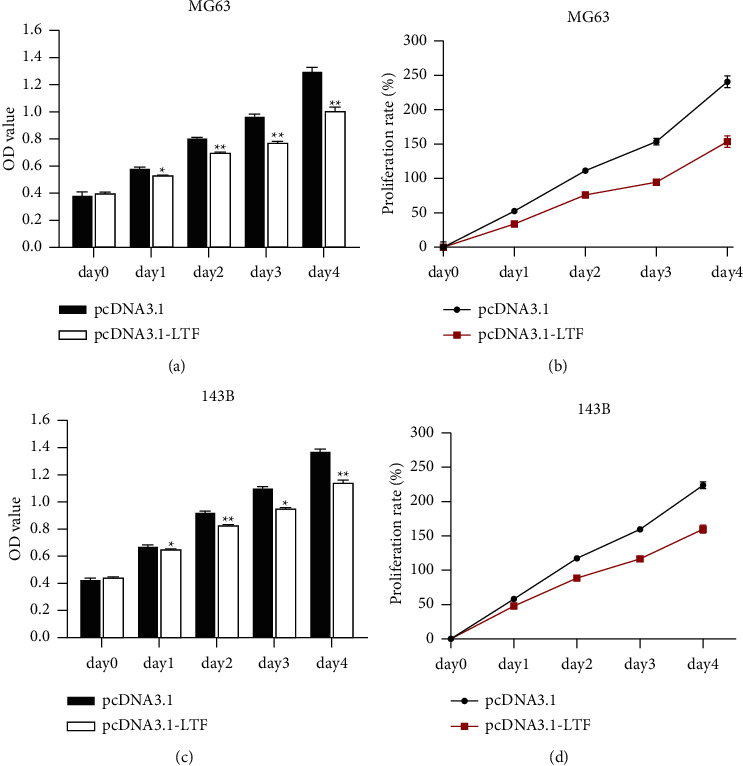
The effect of LTF on the proliferation of osteosarcoma cells. (a) The OD value of MG63 cells. (b) The proliferation rate of MG63 cells. (c) The OD value of 143B cells. (d) The proliferation rate of 143B cells. ^*∗*^*P* < 0.05, ^*∗∗*^*P* < 0.01 vs. pcDNA3.1, *n* = 3.

**Figure 7:**
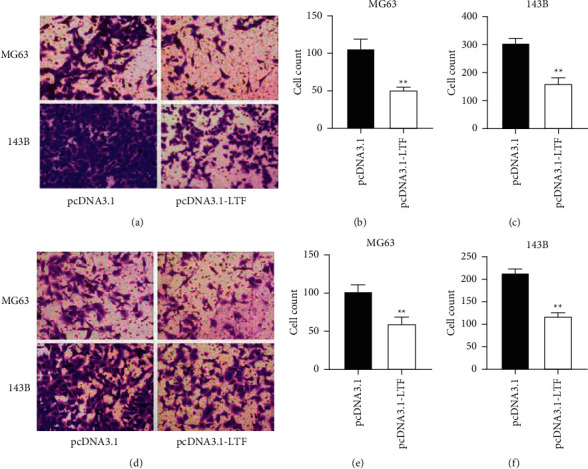
The effect of LTF on migration and invasion of osteosarcoma cells. (a) Representative images of cell migration assay. (b) The effect of LTF on the migration of MG63 cells. (c) The effect of LTF on the migration of 143B cells. (d) Representative images of cell invasion assay. (e) The effect of LTF on the invasion of MG63 cells. (f) The effect of LTF on the invasion of 143B cells. ^*∗∗*^*P* < 0.01 vs. pcDNA3.1, *n* = 3.

The CCK-8 assay showed that the OD value in the LTF overexpression group was lower than that in the pcDNA3.1-transfected group (Figures 6(a) and 6(c)). Therefore, the overexpression of LTF inhibited the proliferation of MG63 and 143B cells (Figures 6(b) and 6(d)). Transwell migration and invasion assays showed that the overexpression of LTF inhibited the migration and invasion ability of MG63 and 143B cells (Figure 7).

## 4. Discussion

Osteosarcoma is the commonest primary bone malignant tumor with a high rate of metastasis [13]. There is still a need for specific prognostic biomarkers and drug targets for osteosarcoma. Identifying specific prognostic biomarkers may contribute to the clinical management of osteosarcoma.

In the present study, we collected osteosarcoma-associated gene profiles in the GEO database and screened the top 5000 genes from GSE32981 using MAD. A weighted gene coexpression network was constructed, and the modules of this network were calculated by TOM. The relevance between eigengenes of 18 modules and origin, age, gender, and metastases status of osteosarcoma samples was calculated. The results showed that the tan module and midnight blue module are correlated with origin and metastases of osteosarcoma, respectively. The origin and metastases status of osteosarcoma were related to the response to therapies and may influence clinical therapeutic decision making [14, 15]. Therefore, these two modules were selected for further investigation.

We then performed enrichment analysis using Metascape. Genes involving in the tan module were mainly enriched in osteosarcoma-related biological processes including “regulation of peptidase activity” [16–18], “phenylalanine metabolism” [19, 20], and “regulation of growth” [21, 22]. Also, genes involving in the midnight blue module were mainly enriched in osteosarcoma-related biological processes, including “DNA damage/telomere stress-induced senescence” [23–25], “metalloprotease DUBs” [26–28], and “interferon alpha/beta signaling” [29–31].

In addition, the correlation among these genes was calculated by Spearman correlation analysis. Gene pairs with |SCC| > 0.6 were identified to be screened to construct the coexpression network with 160 nodes and 177 edges. The hub nodes with high degree from the network were identified in the tan module and midnight blue module. Kaplan–Meier analysis of these key genes was performed. Among the 29 genes, four of them was correlated with the prognosis of osteosarcoma patients. C10orf107, HIST1H2AK, and NEXN were from the midnight blue module, and LTF was from the tan module. The expression of C10orf107 and NEXN was positively correlated with poor survival of osteosarcoma patients, whereas the expression of HIST1H2AK and LTF was negatively correlated with poor survival of osteosarcoma patients.

The effect of C10orf107 in cancer was poorly understood, and only a rare translocation t(3; 10) (q26; q21) was observed in an acute myeloid leukemia patient, presented as a fusion of MECOM (chromosome 3q26.2) and C10orf107 (chromosome 10q21.2) [32]. NEXN (encode Nexilin) is related to cardiovascular diseases, including hypertrophic cardiomyopathy, coronary artery disease, and septal defects [33–35]. However, its role in cancer has not been reported.

LTF has been considered as a tumor suppressor in multiple cancers. LTF is deficient or lowly expressed in prostate cancer, nasopharyngeal carcinoma, oral squamous cell carcinoma, etc. [36–38]. Osteosarcoma patients with lower LTF have a poor survival rate compared with those with higher LTF (Figure 5). However, its effect on osteosarcoma is still not known. We then detected its effect on osteosarcoma cells. The results showed that the overexpression of LTF inhibited the proliferation of osteosarcoma cells (Figure 6). Moreover, the upregulation of LTF also promoted the migration and invasion of osteosarcoma cells (Figure 7). These results were in accordance with the view that LTF is a tumor suppressor. LTF has inhibitive effects on cancer progression. Xiao et al. revealed that the growth inhibitory effects of LTF were through a p27/cyclin E-dependent pathway in head and neck cancer cells [39]. Similarly, Zhang et al. found that lactoferrin exerts an inhibitive effect on breast cancer cell growth through inducing cell cycle arrest, with little effect on normal breast cancer cells [40]. LTF also showed migration and/or invasion inhibitory effect in different types of cancer cells.

LTF could inhibit the migration of colorectal adenocarcinoma cells (Caco-2) and gastric adenocarcinoma cells (AGS) [41]. LTF suppressed and even reversed epithelial-to-mesenchymal transition process in oral squamous cell carcinoma [42] and glioblastoma [43]. Moreover, the administration of LTF inhibited the liver and spleen metastasis of L5178Y-ML25 cells and lung metastasis of L5178Y-ML25 cells [44]. A recent study found that LTF deficiency enhanced lung metastasis of melanoma in an LTF KO mouse model, which was related to the enhancing of the TLR9 pathway [30].

In summary, osteosarcoma-related datasets from the GEO database had been systematically analyzed. WGCNA showed that the tan module and midnight blue module are highly correlated with origin and metastases of osteosarcoma, respectively. Enrichment analyses showed that genes in these two modules were associated with cancer-related pathways. A gene coexpression network was constructed using these genes, and the key genes were identified with a high degree. Four key genes (C10orf107, HIST1H2AK, NEXN, and LTF) were found to be correlated with the prognosis of osteosarcoma patients. LTF has the highest AUC value, and its inhibitive effect on osteosarcoma cells was validated. Still, further studies are needed to reveal the precise effect and mechanism of these genes.

## 5. Conclusions

The key gene we identified, such as LTF, could be a prognosis biomarker and therapeutic drug target for osteosarcoma. Our study brought new insights into the investigation of osteosarcoma.

## Figures and Tables

**Table 1:** The top 10 hub genes in the tan module.

Gene name	Class	Degree
MLPH	tan	15
ITGA8	tan	9
MGC34132	tan	8
C6orf165	tan	7
ANXA8	tan	7
CYB5	tan	6
FLJ14146	tan	6
CRY2	tan	4
CFL2	tan	4
MPP6	tan	3
EPHA3	tan	3
LTF	tan	3
FLJ10204	tan	3
CLDN4	tan	3
SERPINA3	tan	3
JMJD1C	tan	3
C9orf99	tan	3
FLJ20421	tan	3
NR4A1	tan	3

**Table 2:** The top 10 hub genes in the midnight blue module.

Gene name	Class	Degree
FLJ25286	Midnight blue	9
ITPR3	Midnight blue	8
HIST1H2AK	Midnight blue	6
HIST1H1E	Midnight blue	6
NEXN	Midnight blue	6
HIST1H2AB	Midnight blue	6
COPS5	Midnight blue	5
SLC35B1	Midnight blue	5
HIST1H1C	Midnight blue	5
C10orf107	Midnight blue	5

## Data Availability

The data that support the findings of this study are available from the corresponding author upon request.

## Additional Points

*Key Messages*. (1) The tan module and midnightblue module are with the highest correlation with origin and metastases of osteosarcoma, respectively. (2) Overexpression of LTF promoted the proliferation, migration, and invasion of osteosarcoma cells. (3) Key genes including LTF, C10orf107, HIST1H2AK, and NEXN may serve as prognostic biomarkers for osteosarcoma.

## Supplementary Materials

Supplementary Materials Figure S1: the expression of LTF in osteosarcoma samples and normal controls. (A) The expression of LTF in osteosarcoma samples and normal controls calculated using the GSE36001 dataset. (B) The expression of LTF in osteosarcoma samples and normal controls calculated using the GSE99671 dataset.
