# Supplementary figures and images for: Identification of LTF as a Prognostic Biomarker for Osteosarcoma
Source: J Oncol. 2022 Jan 21;2022:4656661. doi: 10.1155/2022/4656661 (PMC8799371; doi:10.1155/2022/4656661)

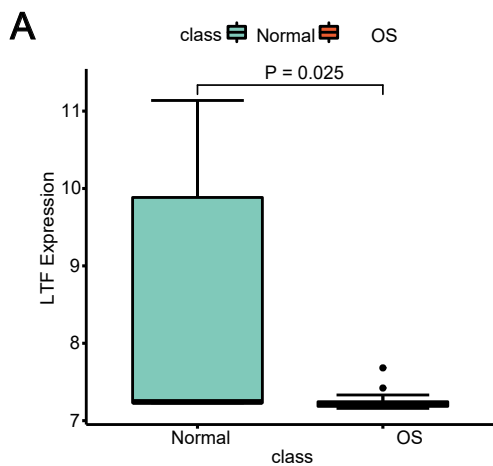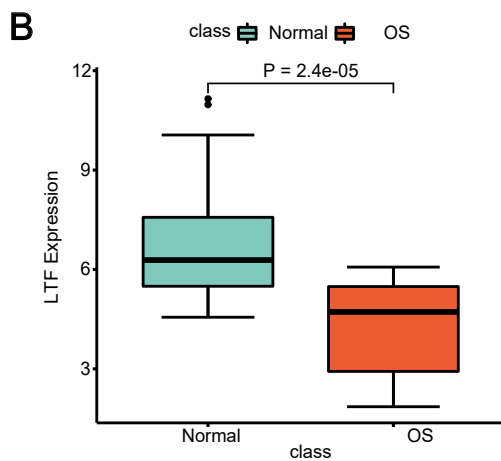

Supplement: Supplementary Materials — Figure S1: the expression of LTF in osteosarcoma samples and normal controls. (A) The expression of LTF in osteosarcoma samples and normal controls calculated using the GSE36001 dataset. (B) The expression of LTF in osteosarcoma samples and normal controls calculated using the GSE99671 dataset. [file 4656661.f1.pdf]
